# Age-related hearing loss was accelerated by apoptosis of spiral ganglion and stria vascularis cells in ApoE KO mice with hyperglycemia and hyperlipidemia

**DOI:** 10.3389/fneur.2022.1016654

**Published:** 2022-11-03

**Authors:** Phuong Thi Thanh Nguyen, Hayoung Song, Boyoung Kim, Yoo Yeon Kim, Chulho Kim, Jun Ho Lee, Jun Gyo Suh

**Affiliations:** ^1^Department of Medical Genetics, College of Medicine, Hallym University, Chuncheon, South Korea; ^2^Institute of New Frontier Research, Hallym University College of Medicine, Chuncheon, South Korea; ^3^Department of Neurology, Chuncheon Sacred Heart Hospital, Hallym University, Chuncheon, South Korea; ^4^Department of Otorhinolaryngology-Head and Neck Surgery, College of Medicine, Hallym University, Chuncheon, South Korea; ^5^Institute of Medical Science, College of Medicine, Hallym University, Chuncheon, South Korea

**Keywords:** age-related hearing loss, spiral ganglion neuron, stria vascularis, atherosclerosis, apoptosis, hyperglycemia, hyperlipidemia

## Abstract

Age-related hearing loss (ARHL) is associated with diabetes and/or dyslipidemia in humans. However, the detailed mechanism for the development of ARHL by diabetes and/or dyslipidemia has not been elucidated. In this study, we investigated the etiology of ARHL in apolipoprotein E (ApoE)-deficient mice with diabetes and dyslipidemia. The atherosclerotic CD-STZ (mice fed with a control diet and received an STZ injection), WD-con (mice fed with a western diet), and WD-STZ (mice fed with a western diet and received an STZ injection) mice showed a 2.4-, 4.9-, and 6.8-fold larger area, respectively, occupied by lesions throughout the aorta compared with the CD-con mice. A significantly larger area under the curve (AUC) was observed in the STZ-treated groups than in the non-treated groups based on the oral glucose tolerance test (OGTT). At 20 weeks of age, HbA_1c_ levels were significantly higher in the CD-STZ and WD-STZ mice than in the CD-con and WD-con mice. In all the groups, the auditory brainstem response (ABR) thresholds of the 16-week-old mice were significantly higher compared with those of the 8-week-old mice. In particular, in the WD-STZ mice, the ABR thresholds of the left and right ears reached the maximum decibel peak equivalent sound pressure levels (130 dBpeSPL), which is a sign of deafness. The apoptotic spiral ganglion neurons (SGNs) of the WD-STZ mice were significantly increased compared with those of the other three groups, indicating that SGN apoptosis resulted in hearing loss in STZ-induced diabetic ApoE KO mice fed with a WD. A significant loss of the stria vascularis cells was observed in the WD-STZ group compared with the CD-con mice. In the organ of Corti, few apoptotic hair cells were found in all the groups; however, no significant difference was observed. Therefore, we consider that the reduced hearing ability in the STZ-treated and WD-fed groups was attributed to the damage to the SGN and stria vascularis in the cochlea. Thus, our results indicated that ototoxicity by diabetes and/or dyslipidemia accelerated ARHL in ApoE KO mice, thereby suggesting the importance of appropriate treatment of patients with diabetes and/or dyslipidemia to prevent ARHL.

## Introduction

According to a recent report by the World Health Organization, hearing loss has gradually become a common health problem that occurs in 466 million people around the world. The estimated number of people with hearing impairment by 2050 will be over 900 million, which means one in every ten people would probably encounter this disability (1). Among various types of hearing loss, age-related hearing loss (ARHL) is getting more attention as its etiology has not been confirmed; moreover, no effective treatment and prevention methods have been recorded. Thus, several studies have been performed to discover its underlying etiology and pathophysiology. There are three controversial hypotheses concerning the genesis of this hearing impairment. First, the sensory hypothesis suggests that the main destructive cause is the degeneration of cochlear hair cells. The second hypothesis, which focuses on the metabolic origin, claims that atrophy of the stria vascularis (SV) constitutes a loss of nutritional support for the cochlea. Third, the neuronal hypothesis accounts for the degeneration of the spiral ganglion neurons (SGNs) that connect the cochlea and the auditory cortex in the brain (2–4).

Risk factors for the development of ARHL are genetic, heredity, oxidative stress, noise exposure, ototoxic drugs, and vascular degeneration (5–9). The cochlea has a single arterial supply system with few collateral vessels; thus, this structure is fragile and is considered highly sensitive to vascular changes (10). Recently, atherosclerosis, diabetes mellitus (DM), dyslipidemia, and hypertension have been reported to promote vascular degeneration (11–17). DM is found to correlate with the prevalence of hearing losses at high frequencies in clinical studies (18, 19), and streptozotocin (STZ)-induced DM impairs the auditory pathway in mouse models (20–23). However, the pathophysiological mechanisms that DM affects ARHL have not been fully elucidated and are contradictory. Many studies demonstrated the decrease of SGNs to be attributed to hyperglycemia, which leads to hearing impairment (19, 24, 25). Disrupted microcirculation is supposed to induce a loss of SGNs owing to the inadequate supply of oxygen and nutrients for the cochlea (26). On the contrary, some studies suggest that the decline of the outer hair cells is the major change in the cochlea of patients with diabetes and no significant difference exists in the number of spiral ganglion cells (14, 27). Moreover, the SV reportedly demonstrated decreased thickness and loss of intermediate cells in another study (28).

In our previous study, apoptosis of the SGNs appeared to be the main problem in ARHL of apolipoprotein E knockout (ApoE KO) mice with atherosclerotic development and hyperlipidemia (12). With the additional progression of DM in the same mouse model, we hypothesized that the SGNs are primarily and majorly degenerated, followed by the SV. Specifically, ApoE KO mice served as the animal model for hyperlipidemia and atherosclerosis and were continuously fed with a Western diet (WD) for the rapid development of these diseases. In addition, we induced diabetes through a streptozotocin (STZ) injection. The chronological progressions of diabetes, hyperlipidemia, atherosclerosis, and hearing loss were determined and analyzed. Our findings suggest that the incorporation of hyperglycemia with hyperlipidemia and atherosclerosis could exacerbate ARHL in ApoE KO male mice.

## Methods

### Animals

**Figure 1:**
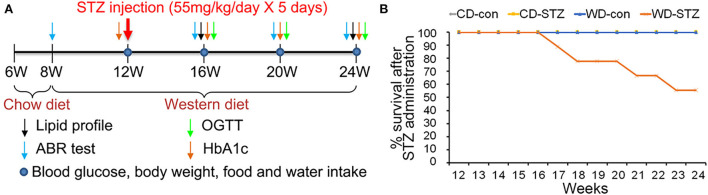
Experimental protocol and survival rate. **(A)** Experimental protocol, including procedures and timeline, for each group. A western diet (WD) was provided at 8 weeks of age, and streptozotocin (STZ) was injected at 12 weeks of age to induce hyperlipidemia and hyperglycemia. **(B)** Decreased survival rate in the WD-STZ mice due to severe atherosclerosis and diabetes. The WD-STZ mice exhibit a gradual decrease in survival rate starting from 17 weeks of age.

Thirty-five ApoE KO male mice (B6.129P2-Apoe^tm1Unc^/J) were obtained at the age of 6 weeks and were maintained on a chow diet (CD; D12450B, Research Diets Inc., New Brunswick, NJ, USA) containing 10 kcal% fat and 0.0014% cholesterol for 2 weeks (Figure 1A). At 8 weeks of age, the mice were randomly assigned to two groups: Group 1 was continuously fed a CD and group 2 was fed a WD (D12079B; Research Diets Inc., New Brunswick, NJ, USA) containing 41 kcal% fat and 0.125% cholesterol for 16 weeks. At 12 weeks of age, the animals received intraperitoneal injections of citrate buffer (pH 4.5) or STZ (Sigma, St. Louis, MO, USA) at a concentration of 55 mg/kg/day for 5 consecutive days to induce diabetes. Two weeks after STZ injection, mice with fasting blood glucose (FBG) levels > 200 mg/dL were enrolled in the experiment as diabetic mice. Overall, the experimental groups were categorized as follows: (1) mice fed with a CD and received a citrate injection (CD-con, *n* = 7); (2) mice fed with a CD and received an STZ injection (CD-STZ, *n* = 8); (3) mice fed with a WD and received a citrate injection (WD-con, *n* = 7); and (4) mice fed with a WD and received an STZ injection (WD-STZ, *n* = 9). Mice were housed singly (13 ×24× 12 cm, MJ Ltd., Seoul, South Korea) in a specific pathogen-free animal care facility that maintained a temperature of 22 ± 2°C, 55 ± 10% relative humidity, and a 12-h light–dark cycle. Animal experiments were conducted according to the guidelines of the “Institutional Animal Care and Use Committee” of Hallym University (Hallym2018-55).

### Measurement of lipid profiles/glucose level

Total cholesterol (TC) and low-density lipoprotein (LDL) levels were measured in the mice using standard blood chemistry equipment (Kornelab20XT, Thermo Fisher Scientific, Waltham, MA, USA) at 16 and 24 weeks of age. To measure the FBG levels at 12, 16, 20, and 24 weeks, the mice fasted for 6 h and thereafter the blood was drawn from the retro-orbital plexus by using a capillary tube. The blood was subsequently loaded to the blood glucose monitoring meter (Accu-Chek, Roche, San Francisco, CA, USA). Oral glucose tolerance test (OGTT) was performed at 16, 20, and 24 weeks to assess glucose tolerance. In brief, the FBG level was assessed after fasting for 6 h. The mice then received 2 g/kg body weight of D-glucose (Sigma). At 30, 60, and 120 min after glucose administration, the blood glucose was measured using a blood glucose monitoring meter. Lastly, to determine the presence of glucose tolerance, the area under the curve (AUC) was calculated using trapezoidal integration. Hemoglobin A_1c_ (HbA_1c_) levels were measured using an HbA_1c_ analyzer (HLC-723G7, Tosoh, Tokyo, Japan) at 12, 16, 20, and 24 weeks.

### Pancreatic and aortic changes

After 1-day paraformaldehyde (PFA) fixation and paraffin embedding, the pancreatic tissues were sliced into 5-μm sections using an automatic rotary microtome (Reichert-Jung, Wetzlar, Germany). The gross morphology of the pancreatic tissue was determined using hematoxylin and eosin staining.

For pancreatic immunofluorescence evaluation, sectioned tissue slides were deparaffinized and dehydrated. Next, the tissue sections were permeabilized in 0.03% TBST, washed with 1× Tris-buffered saline (TBS) for 5 min, and blocked with 10% horse serum (HS) in TBS for 40 min. Insulin and glucagon expression in the islets was detected upon incubation with anti-insulin antibody (1:1000, Santa Cruz Biotechnology Inc., Dallas, TX, USA) and anti-glucagon antibody (1:100, Santa Cruz Biotechnology Inc.), respectively, in 10% HS in TBS overnight at room temperature. Subsequently, on the following day, the sections were washed and incubated with Alexa Fluor 594 conjugated donkey anti-rabbit immunoglobulin G secondary antibody (1:1000, Abcam, Cambridge, UK) and Alexa Fluor 488 conjugated donkey anti-goat immunoglobulin G secondary antibody (1:2000, Abcam) for 2 h. The sections were washed and mounted using the ProLong^TM^ Gold Antifade Mountant (Invitrogen, Waltham, MA, USA). Fluorescence microscopy (Carl Zeiss, Oberkochen, Germany) was used to capture the images. Insulin and glucagon expression in the pancreas was measured using ImageJ software (National Institutes of Health, Bethesda, MD, USA).

After *in situ* perfusion with phosphate-buffered saline (PBS) followed by 4% paraformaldehyde, the aorta, including the aortic arch and aortic iliac, was isolated from the mice at 24 weeks of age. All perivascular and adipose tissues surrounding the aortas were removed, and the aortas were longitudinally opened and pinned flat in a black silicon-attached Petri dish. They were then incubated in a 4% paraformaldehyde–sucrose solution overnight at room temperature. The tissues were subsequently rinsed three times for 10 min with 1× PBS and 70% ethanol for 5 min, stained with 5% Sudan IV (Sigma, USA) in 100% acetone and 70% ethanol (1:1) for 10 min, decolorized two times for 3 min with 70% ethanol, and washed for 5 min with 1× PBS to remove the ethanol. A stereoscopic microscope (SMZ745T, Nikon, Tokyo, Japan) was used to acquire *en face* images of the aortas. The atherosclerotic plaque areas were measured using ImageJ software (National Institutes of Health).

### Auditory brainstem response (ABR) test

To evaluate the auditory function, the ABR thresholds were measured in all the mice at 8, 16, 20, and 24 weeks of age. Anesthesia was induced with 3% isoflurane mixed with oxygen using the RC2+ anesthesia system (Labs Inc., Centennial, CO, USA). Click sounds were generated using ECLIPS (Interacoustics, Middelfart, Denmark), and the output was recorded using OtoAccess software (version 1.4, Interacoustics). Sound responses were recorded to dBpeSPL in the left and right ears. The auditory thresholds were determined by increasing the sound intensities of the click stimulus from 30 to 130 dBpeSPL.

### Histological analysis of the cochlea

After a 1-day PFA fixation, the cochleae were incubated in decalcifying solution-lite (Sigma) for 5 h at room temperature. After processing and embedding, the paraffin-embedded cochlear tissues were sectioned into 7-μm sections. The morphology of the cochlear sections was determined using hematoxylin and eosin staining. The DeadEnd™ Fluorometric TUNEL System (Promega, Madison, WI) was used to conduct the TUNEL assay. Photographs were taken in the left and right ears at the cochlear turn using a confocal microscope (LSM 710, Carl Zeiss). The expression intensity was measured using Zen Blue (Carl Zeiss) and ImageJ software (National Institutes of Health).

Immunofluorescence staining with myosin VIIA antibody was conducted to evaluate the morphology of the hair cells. First, the 7-μm sectioned cochlear slides were deparaffinized, dehydrated, and permeabilized in 0.025% TBST followed by washing and blocking with 1% bovine serum albumin and 10% HS in TBS for 1 h. The sections were subsequently incubated with an anti-myosin VIIA antibody (1:100, Abcam) in 1× TBS for 16 h at 4°C. Thereafter, the sections were washed and incubated with Alexa Fluor 488 conjugated goat anti-rabbit immunoglobulin G secondary antibody (1:500, Abcam) for 2 h at room temperature. The sections were washed, stained with DAPI (Invitrogen), and mounted using the ProLongTM Gold Antifade Mountant (Invitrogen). Fluorescence microscopy (Carl Zeiss) was used to capture the images. The expression of insulin and glucagon in the pancreas was measured using ImageJ software (National Institutes of Health).

### Statistical analysis

All the data are expressed as mean ± standard deviation. Data were analyzed by the Student's t test using IBM SPSS Statistics 22 (SPSS Inc., USA). Statistical significance was set at ^*^ *p* < 0.05, ^**^ *p* < 0.01, ^***^ *p* < 0.001, and ^****^ *p* < 0.0001.

## Results

### Experimental procedure and mice survival rate

The induction and examination of hyperglycemia and hyperlipidemia, as well as the validation of ARHL progression, were longitudinally performed (Figure 1A). In the CD-con, CD-STZ, and WD-con groups, all the mice showed good health conditions until the end of the experimental procedure (24 weeks of age). However, approximately 46% of the mice in the WD-STZ group suffered from a serious health condition with more than 30% weight loss; thus, they were killed before the schedule (Figure 1B).

### Hyperlipidemia induction and atherosclerotic plaque development

**Figure 2:**
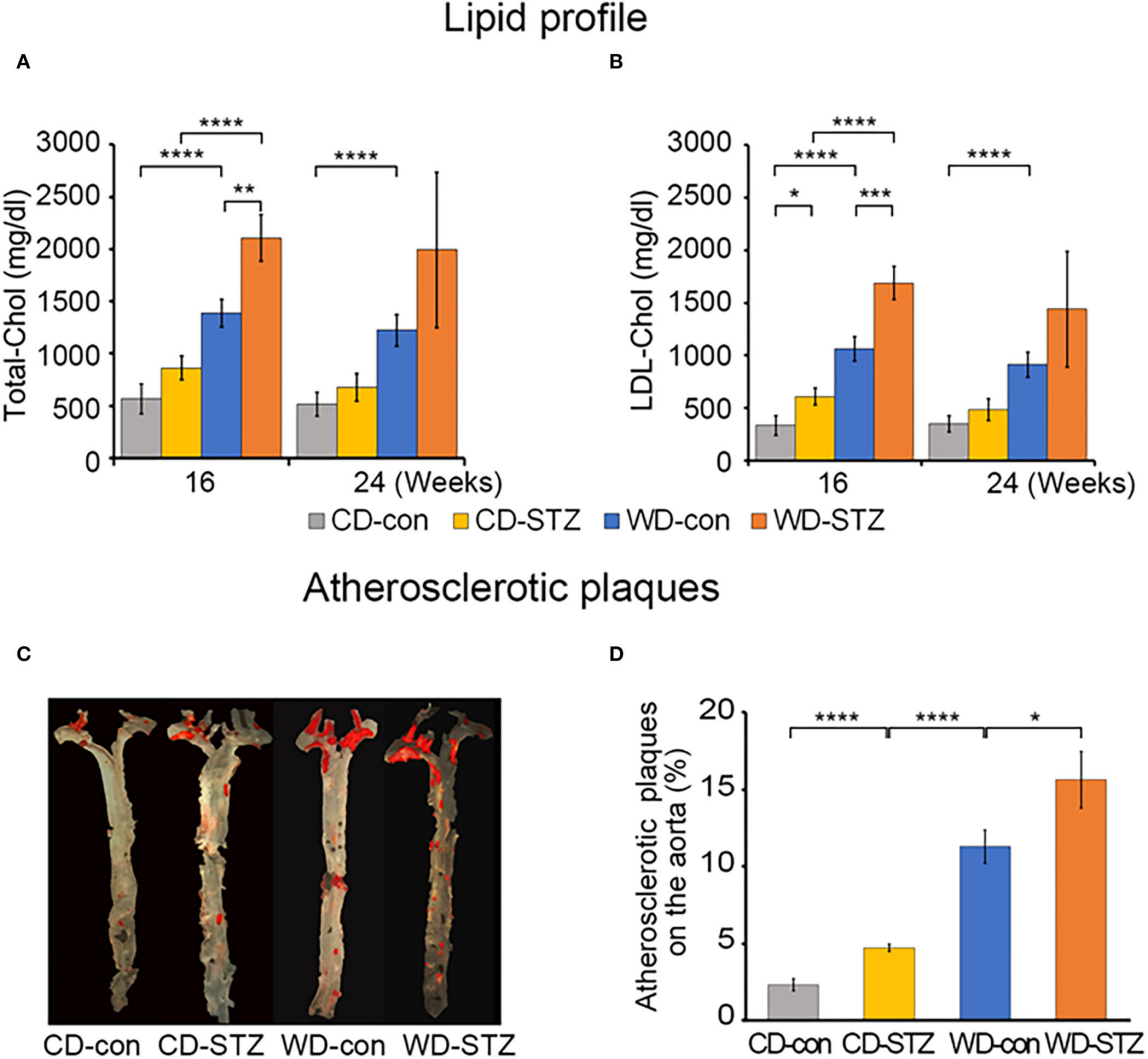
Lipid profiles and atherosclerotic plaques. **(A)** The plasma total cholesterol level in each group of mice at 16 and 24 weeks of age. **(B)** Low-density lipoprotein (LDL) cholesterol in each group of mice at 16 and 24 weeks of age. **(C)** Representative *en face* images of the atherosclerotic plaques in the whole aorta using Sudan IV staining (red). **(D)** Percentage of lesion areas relative to the whole aortic area in CD-con, CD-STZ, WD-con, and WD-STZ ApoE KO male mice at 24 weeks of age. * *p* < 0.05, ** *p* < 0.01, *** *p* < 0.001, and **** *p* < 0.0001.

We measured the total cholesterol (TC) and low-density lipoprotein (LDL) cholesterol at 16 and 24 weeks of age. TC and LDL cholesterol levels showed a consistent trend among the four experimental groups in the 16- and 24-week-old mice. TC and LDL cholesterol levels, ranging from the highest to the lowest, were WD-STZ > WD-con > chow diet (CD)-STZ > CD-con, with significant differences between the pairs of the 16-week-old mice (Figures 2A,B). However, at 24 weeks of age, there was no marked difference between the CD-con and CD-STZ groups and between the WD-con and WD-STZ groups. Owing to the loss in the number of mice, the WD-STZ mice did not exhibit any significant differences compared with the mice of the other three groups. A significant increase was observed only in the TC and LDL of the WD-con mice compared with those of the CD-con mice.

To investigate the influence of a WD (high-fat diet) and STZ-induced diabetes on atherosclerosis in ApoE KO male mice, we performed *en face* staining of the aorta with Sudan IV (Figure 2C). In all the groups, the lesions were localized mostly in the ascending aorta, aortic arch, and its branches, especially the brachiocephalic artery. The atherosclerotic CD-STZ, WD-con, and WD-STZ mice showed a 2.4-, 4.9-, and 6.8-fold larger area, respectively, occupied by lesions throughout the aorta compared with the CD-con mice (4.7 ± 0.5, 11.3 ± 2.1, 15.6 ± 3.7 vs. 2.3 ± 0.8%, respectively) (Figure 2D). These data demonstrate that a high-fat diet associated with STZ treatment accelerated atherosclerosis in ApoE KO male mice.

### Hyperglycemia induction

**Figure 3:**
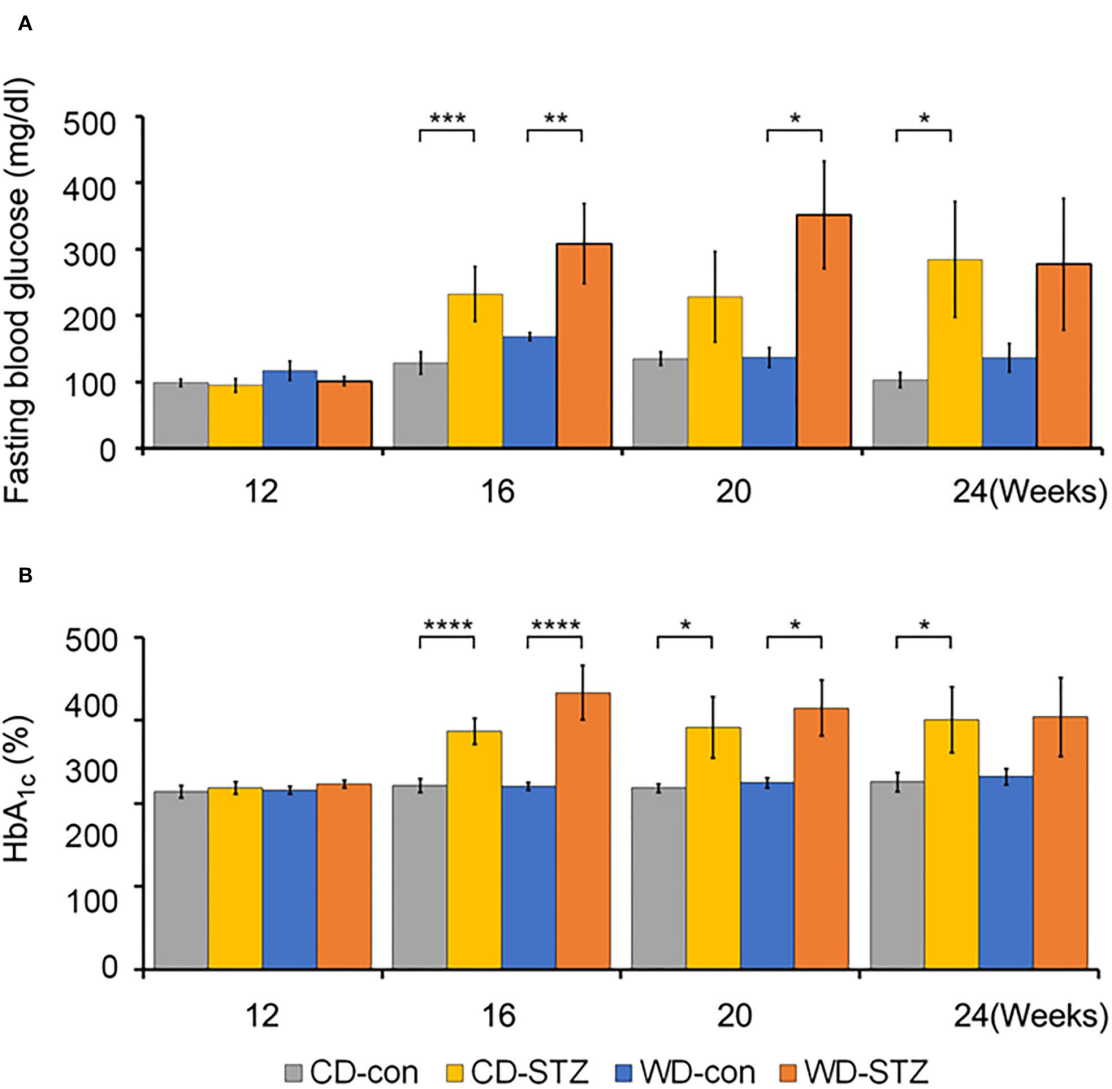
Fasting blood glucose and hemoglobin A_1c_ (HbA_1c_) levels. **(A)** Increased fasting blood glucose levels in STZ-treated mice at 16 weeks of age. Blood glucose levels were checked in all groups after 6 hours of fasting. **(B)** Hemoglobin A1c (HbA_1c_) levels in all groups of mice at 16, 20, and 24 weeks of age. * *p* < 0.05, ** *p* < 0.01, *** *p* < 0.001, and **** *p* < 0.0001.

**Figure 4:**
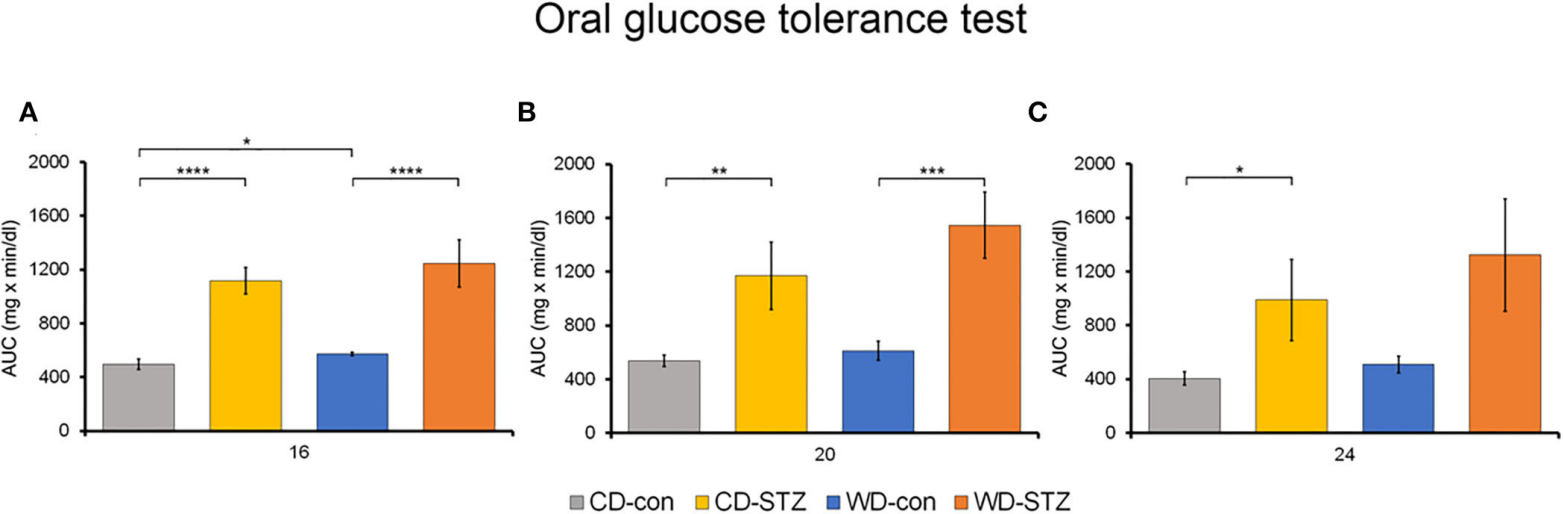
Oral glucose tolerance test (OGTT). The area under the curve (AUC) after the OGTT was calculated in mice at 16 **(A)**, 20 **(B)**, and 24 **(C)** weeks of age. * *p* < 0.05, ** *p* < 0.01, *** *p* < 0.001, and **** *p* < 0.0001.

**Figure 5:**
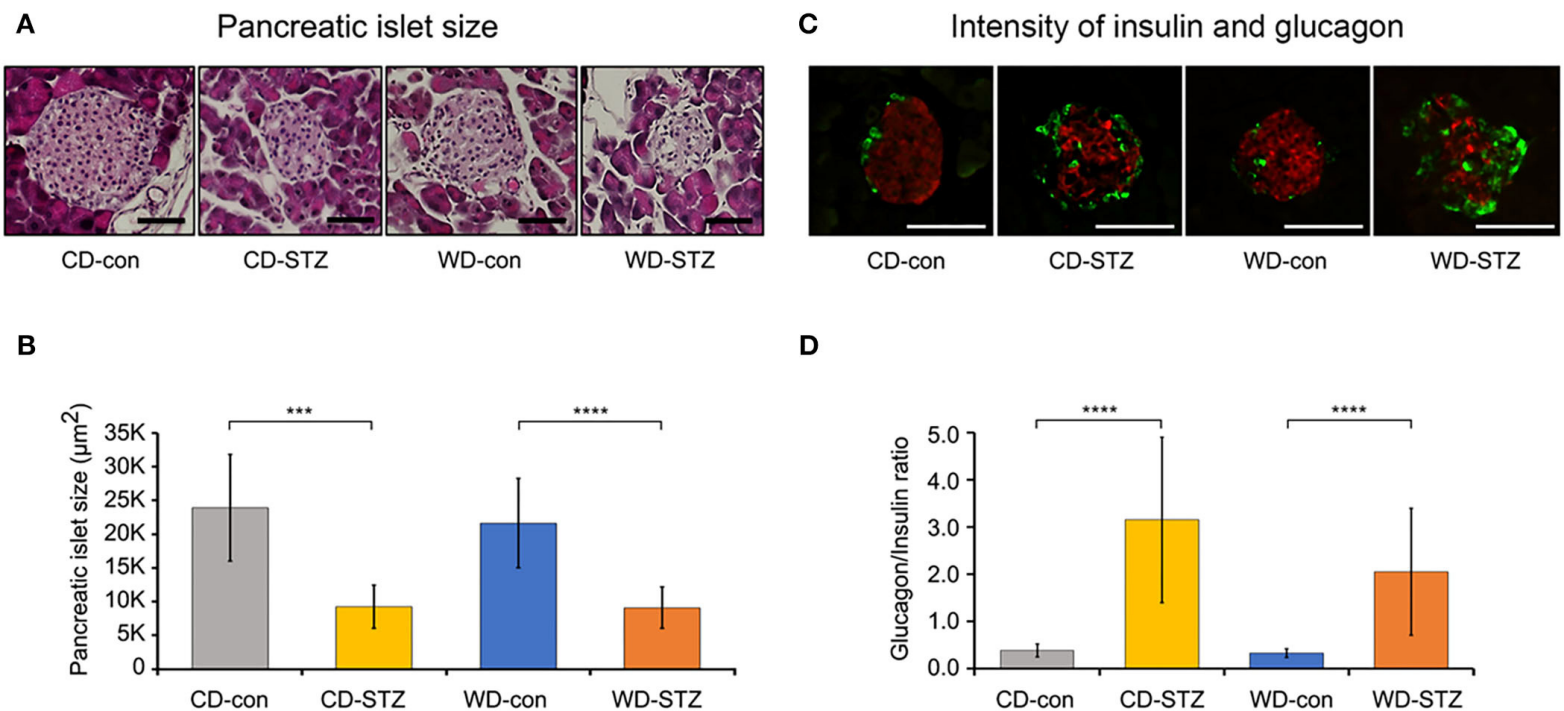
Pancreatic islet size and glucagon/insulin ratio. **(A,B)** Decreased pancreatic islet size in STZ-treated mice confirmed on hematoxylin and eosin staining. **(C,D)** Increased α-cell and decreased β-cell intensities in pancreatic islets of STZ-treated mice. The glucagon (green)/insulin (red) ratio was measured and calculated. *** *p* < 0.001, and **** *p* < 0.0001. Scale bars correspond to 50 μm.

To confirm the induction of diabetes *via* STZ injection in ApoE KO male mice, the FBG measurement, OGTT was performed and the HbA_1c_ levels were evaluated. FBG level, an important indicator of diabetes, was significantly higher in the STZ-treated groups than that in the non-treated groups at 16, 20, and 24 weeks of age (Figure 3A). At 24 weeks of age, the WD-STZ mice did not exhibit a significant increase in the FGB levels compared with the WD-con mice owing to the decreased number of mice in the WD-STZ group at that age. At the same time points, the HbA_1c_ levels were significantly higher in the CD-STZ and WD-STZ mice than in the CD-con and WD-con mice (Figure 3B). In the OGTT conducted at 16, 20, and 24 weeks of age, a significantly larger area under the curve (AUC) was observed in the STZ-treated groups than in the non-treated groups (Figure 4). Notably, the AUC in the WD-con mice was significantly higher than that in the CD-con mice at that age, indicating that high-fat diets can also induce abnormal glucose tolerance in ApoE KO male mice. The pancreatic islets were further examined histologically *via* hematoxylin and eosin staining. Mice in the CD-con and WD-con groups showed a normal pancreatic structure, whereas the pancreas of the CD-STZ and WD-STZ groups showed significant shrinkage of the islets (Figures 5A,B). Furthermore, immunohistological staining of the islets demonstrated that the density of insulin-producing β-cells significantly reduced and the density of the glucagon-producing α-cells significantly increased in the CD-STZ and WD-STZ mice compared with those in the CD-con and WD-con mice (Figures 5C,D). Therefore, STZ injection successfully induced diabetes in ApoE KO male mice with marked increase in the blood glucose levels and modification of the islet's structure.

### Hearing impairment in experimental mice

**Figure 6:**
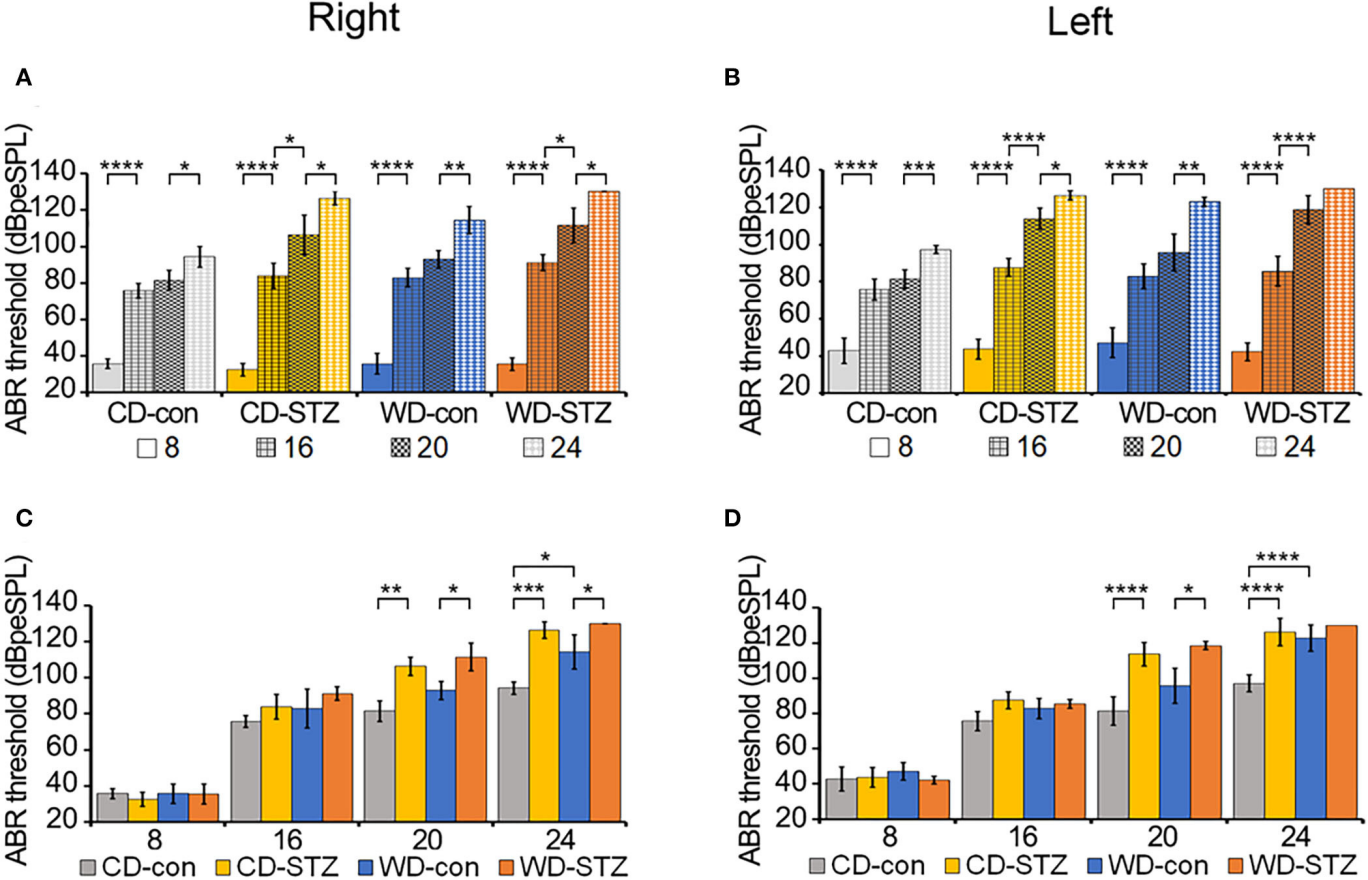
Hearing ability according to age and experimental conditions. **(A,B)** Increased auditory brainstem response (ABR) thresholds with age. ABR thresholds were measured in CD-con, CD-STZ, WD-con, and WD-STZ mice at 8, 16, 20, and 24 weeks of age with click sounds to compare age differences between each group in the right **(A)** and left **(B)** ears. **(C,D)** Increased ABR thresholds due to Western diet (WD) feeding and streptozotocin (STZ) treatment. ABR thresholds were measured in CD-con, CD-STZ, WD-con, and WD-STZ mice at 8, 16, 20, and 24 weeks of age with click sounds to compare differences between groups at the same age in the right **(C)** and left **(D)** ears. * *p* < 0.05, ** *p* < 0.01, *** *p* < 0.001, and **** *p* < 0.0001.

The ABR thresholds were measured in the left and right ears of each group (CD-con, CD-STZ, WD-con, and WD-STZ) at 8, 16, 20, and 24 weeks of age (Figure 6). The ABR thresholds of 16-week-old mice were significantly higher than those of 8-week-old mice, and those of 24-week-old mice were the highest in both ears (Figures 6A,B). In particular, in 24-week-old WD-STZ mice, the ABR thresholds of the left and right ears reached the maximum decibel peak equivalent sound pressure levels (130 dBpeSPL), which is a sign of deafness. These results illustrate that STZ treatment and a high-fat diet were two risk factors for hearing loss progression in ApoE KO male mice.

Alterations in the ABR thresholds between groups at the same age are exhibited in Figures 6C,D. At 24 weeks of age, there were significant differences in both ears between the ABR thresholds of the STZ-treated mice (CD-STZ, WD-STZ) and CD-con mice, indicating that both hyperlipidemia and hyperglycemia affected the hearing ability. Between the CD-con and WD-con mice, the left ears did not display a significant increase in the ABR thresholds until 24 weeks of age. However, significant increase was observed earlier (at 20 weeks) upon comparing the CD-STZ to CD-con and WD-STZ to WD-con mice. These results indicated that the STZ treatment had a greater impact on progressive hearing loss.

### Apoptosis of the SGN and SV cell

**Figure 7:**
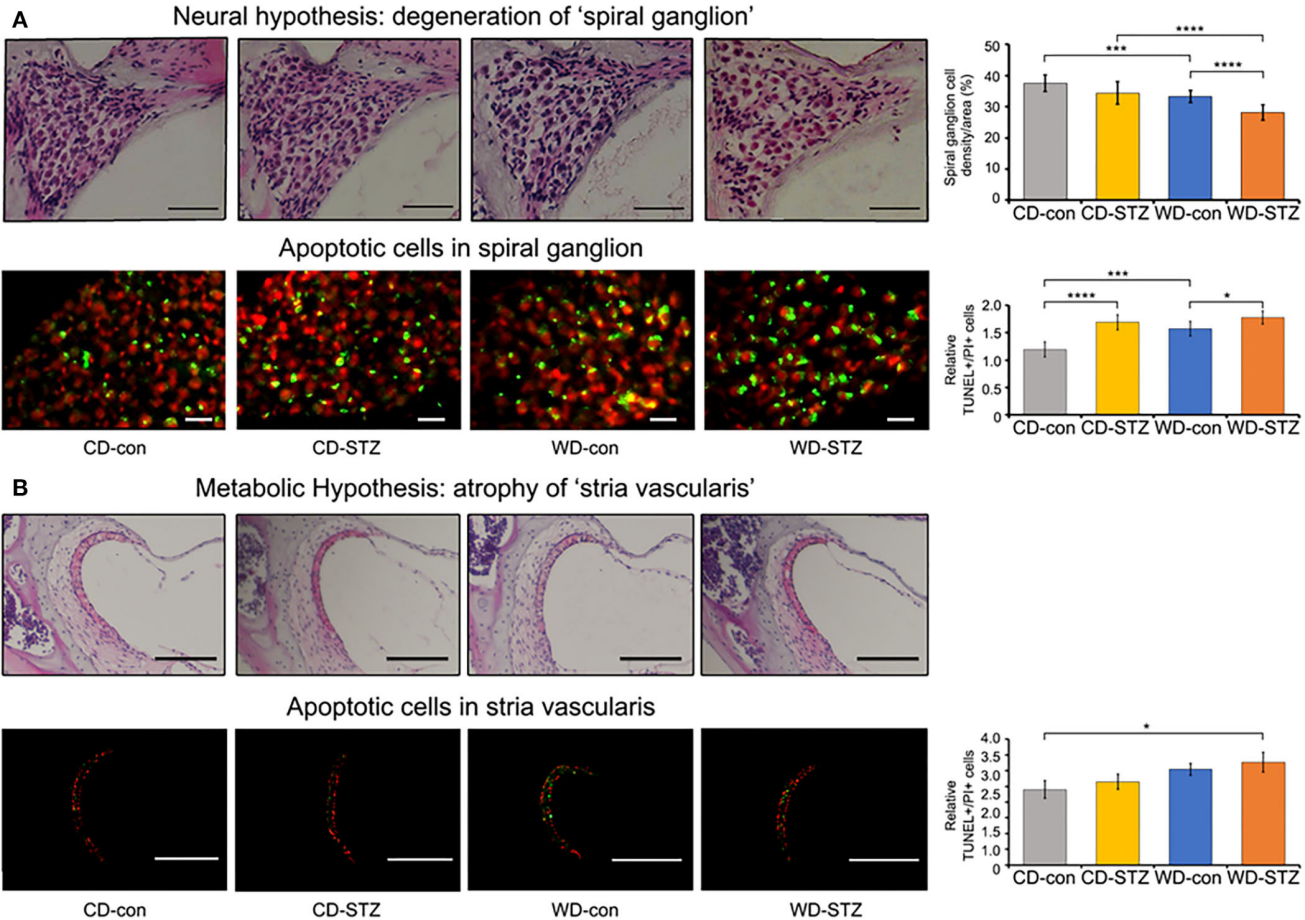
Histological analysis of the cochlear spiral ganglion neurons (SGNs) and stria vascularis. Terminal deoxynucleotidyl transferase dUTP nick end labeling (TUNEL, green) and propidium iodide (PI, red) staining were used to detect apoptosis in the SGNs **(A)** and stria vascularis **(B)** at 24 weeks of age. The TUNEL^+^/PI^+^ ratio of SGNs was significantly different between all groups **(A)**, while that of stria vascularis was significantly different between the CD-con and WD-STZ groups **(B)**. * *p* < 0.05, *** *p* < 0.001, and **** *p* < 0.0001. Scale bars correspond to 50 μm.

**Figure 8:**
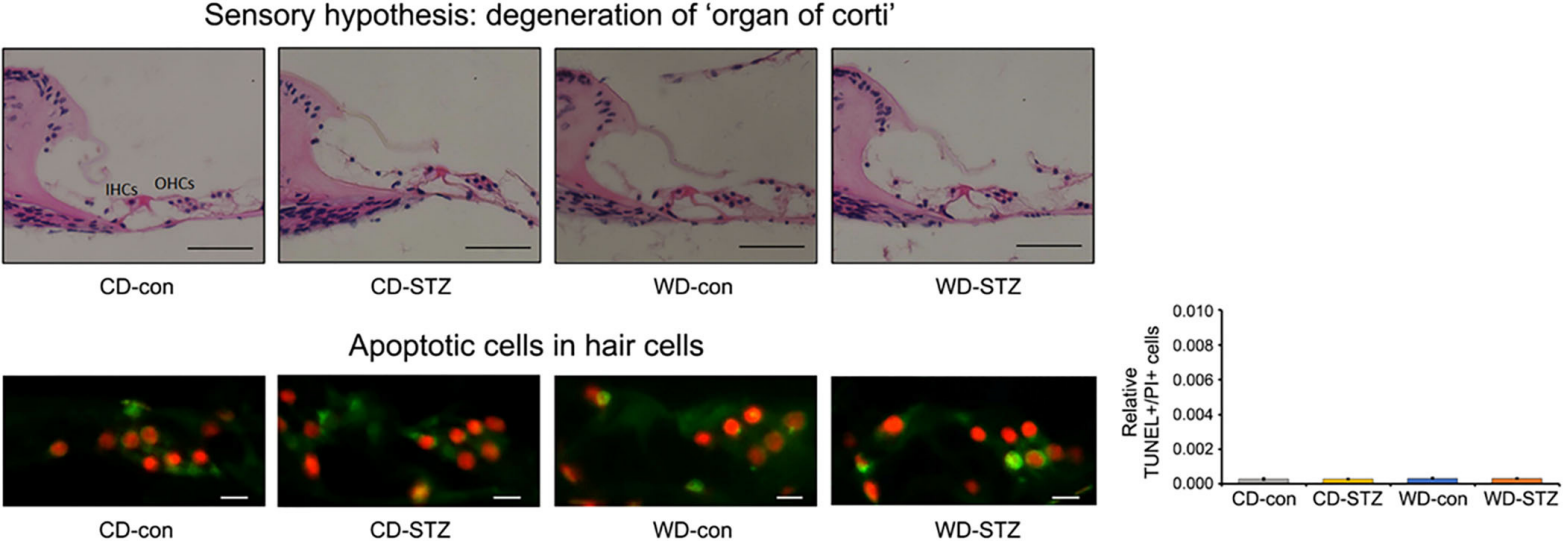
Histological analysis of the organ of Corti. No significant difference was noted in the TUNEL^+^/PI^+^ ratio in hair cells. The average count of apoptosis cells was taken from at least eight cross-sectional images. Scale bars correspond to 50 μm **(upper panel)** and 1 μm **(lower panel)**.

To confirm our hypotheses on the worsening of hearing ability under hyperlipidemic and hyperglycemic states, we first quantified the number of SGNs in the cochlea. A significant loss of SGNs was observed in the WD-STZ group compared with the other three groups. The apoptotic cells (TUNEL-positive cells) in the SGNs of the WD-STZ mice were significantly increased compared with those of the CD-con and CD-STZ mice, indicating that SGN apoptosis resulted in hearing loss in STZ-induced diabetic ApoE KO male mice fed with a WD (Figure 7A). Besides, a significant elevation of apoptotic cells in the SV was observed in the WD-STZ group compared with the CD-con mice (Figure 7B). Finally, we confirmed the normal state of hair cells (Figure 8). Few apoptotic cells with no significant difference were found in the hair cells in all groups. Therefore, we concluded that the reduced hearing ability in the STZ-treated and WD-fed groups was attributed to the damage to the SGNs and SV cells in the cochlea.

## Discussion

Diabetes is a risk factor for hearing impairment in many human studies (29–31). As ARHL may occur earlier among people with diabetes, understanding the pathophysiological mechanisms underlying diabetes and ARHL is essential for managing diabetic hearing damage. Previous reports demonstrate that males with diabetes are more affected by hearing loss than females (31, 32). Therefore, in our study, we only used the ApoE KO male mice as a model for evaluating the relation between STZ-induced diabetes and ARHL. First, the mice were injected with STZ with a concentration of 55 mg/kg/day to induce diabetes. Crucially, HbA_1c_ levels and AUC during OGTT were considerably higher in the CD-STZ and WD-STZ than those in the CD-con and WD-con mice, respectively. These findings are consistent with several previous reports on ApoE KO mice under STZ manipulation (33–37). Moreover, the morphological changes in the pancreatic islets, which contain insulin-producing β-cells and glucagon-producing α-cells, were examined to consolidate the diabetic characteristics. The size of the pancreatic islets of the STZ-treated groups was significantly smaller than that of the non-treated groups. This was expected and is consistent with the results from other studies that used STZ to induce diabetes in mice (38, 39). Moreover, in immunohistochemical analyses, the β-cell masses in the CD-STZ and WD-STZ groups were significantly reduced compared with those in the CD-con and WD-con groups. The α-cell masses and glucagon/insulin ratios in the STZ-treated groups were significantly higher than those in the non-treated groups. These results are quite compatible with the features described in the previous papers (40–42). Considered together, these findings indicate that the ApoE KO male mice model shows the progression of diabetes by STZ injection.

Hyperlipidemia is one of the complications of diabetes (43). Diabetic dyslipidemia is characterized by increase in the cholesterol, triglyceride, and phospholipid levels (44), together with a decrease in the high density lipoprotein cholesterol (45). Thus, diabetes together with a WD could have resulted in the high blood cholesterol levels. In this study, total cholesterol and LDL cholesterol were significantly elevated in WD-fed groups compared with CD-fed groups at 16 weeks of age. Moreover, the cholesterol levels in the CD-STZ and WD-STZ mice were significantly higher than in the CD-con and WD-con mice. These plasma lipid elevations in the STZ-treated groups are consistent with previous reports (33–37). Thus, it is likely that diabetes together with a WD may cause dramatic high cholesterol in the blood.

Diabetic hyperlipidemia plays an important role in the development of atherosclerosis (46). Moreover, approximately 75% of the patients with diabetes reportedly die from atherosclerosis-related cardiovascular diseases (47, 48). Diabetes worsens atherosclerosis by increasing both blood lipid and HbA_1c_ levels, which subsequently results in lipid deposit on the small blood vessels. Additionally, in the presence of diabetes, glycation of LDL cholesterol increases the atherosclerotic plaque in the aorta. These findings show that the atherosclerotic lesions, which were detected by Sudan IV staining, were significantly increased in the STZ-treated groups and WD-fed groups compared with the non-treated groups and CD-fed groups, respectively. Among the four groups, WD-STZ mice developed the most severe atherosclerosis with the largest plaque, which covered approximately 16% of the thoracic aorta. This result is consistent with the abovementioned information.

The association between diabetes and hyperlipidemia causes a sudden increase in the blood viscosity and impairs blood flow (49). The increased plasma lipid can produce lipid deposits in the small arteries near the cochlea and damage the cochlear neural cells, followed by interruption of the auditory signals to the brain. It is well known that a high cholesterol level leads to arteriosclerotic changes in the vessel walls and narrowing of the lumen. Specifically, ischemic damage to the cochlea causes structural and functional disruption (50). Moreover, the reactive oxygen species (ROS) is increased in dyslipidemic conditions, which leads to mitochondrial dysfunction and cellular apoptosis (6, 51). On the contrary, electromotile response of the hair cells could be impaired owing to the increased uptake of cholesterol by hair cells, which increases their stiffness (52). Although many tests exist for evaluating the hearing ability, ABR is one of the most popular tests which provides information about how the inner ear and the auditory nerve respond to sound stimulation (53, 54). In previous studies, the ABR thresholds are higher in patients with diabetes than in the normal group (19, 55). Furthermore, the increase in the ABR thresholds is observed in both type 1 and type 2 diabetic mice (21–23). We also found a significant elevation in the ABR thresholds in the two STZ-treated groups. A marked increase in the ABR threshold was detected between the STZ-treated and non-STZ-treated mice at 24 weeks of age. Notably, in our study, all the groups showed a marked shift in the ABR thresholds at 16 weeks of age. These results are as expected because the allele in the C57BL/6 background of ApoE KO mice is responsible for the early progression of ARHL (56, 57).

There are five factors attributable to the association between diabetes and ARHL, including microangiopathy, advanced glycation end products (AGEs), ROS, down-regulated Na–K−2Cl cotransporter (NKCC) function, and the accumulation of toxic glucose metabolic by-products. First, microangiopathy was claimed to cause complications not only in the auditory pathway but also in the cochlea, which is highly microvascular. Many studies support this theory (19, 24, 25). Disrupted microcirculation is supposed to induce a loss of SGNs owing to the inadequate supply of oxygen and nutrients to the cochlea (26). Second, the formation of AGEs is enhanced in diabetes due to hyperglycemia. Increased glycation of myelin results in decreased auditory synaptic function, finally causing hearing impairment (58). Third, increased ROS, which results from abnormal metabolism of glucose, is a crucial factor that impacts hearing loss (19). Fourth, insulin resistance in type 2 DM is associated with the down-regulation of NKCC1, which is the protein in the epithelial cells of the SV (59). This part plays an important role in maintaining the endocochlear potential; thus, the reduction in the NKCC1 level accelerates ARHL (60, 61). Finally, hyperglycemia induces neural degeneration through the accumulation of the toxic glucose metabolic by-products, which is related to the impairment of the nerve conduction pathway (62). Diabetes-induced hearing loss could be attributed to these five factors. In clinical studies, patients with diabetes experience changes in their cochlea, which results in mild to severe hearing loss. These changes include thickening of the basilar membrane, spiral modular vessels, atrophy of the SV, and loss of OHCs and spiral ligament cells in humans (14, 27, 63, 64). Morphological changes in the organ of Corti have also been examined in various studies using mouse models. The cochlea of the STZ-induced diabetic C57BL/6 mice showed a loss of SGNs and a thickened vessel wall in the modiolus; however, no difference was observed in the IHCs, OHCs, and the lateral wall. In this study, no abnormality was found in the spinal ligament fibrocytes (Figure 7B upper panel) during H&E staining. Hao et al. (65) reported that Sox 10 expression cells in spiral ligament decreased significantly with age in mice and humans (65). Therefore, the function of spinal ligament fibroblasts in our model mice should be confirmed in future. Lee et al. (28) also demonstrated that diabetes causes apoptosis, cell loss, and mitochondria impairment in the SGNs, together with the decrease in the thickness of the SV in the Akita mouse model (28). The study by Lee et al. (23) concluded that hyperglycemia and obesity may promote sensorineural hearing loss in *ob/ob* mice through OHC degeneration and loss of SGNs (23). Based on the neuropathy theory and a recent study conducted by Kim et al. (12), which found evidence of SGN apoptosis in ApoE KO male mice fed with a WD, we hypothesized that the main and first cochlear change in the STZ-induced diabetic ApoE male mice fed with a WD is SGN dysfunction. In fact, we found that the density of cochlear SGNs significantly reduced in the WD-STZ mice compared with the CD-con, CD-STZ, and WD-con mice upon hematoxylin and eosin staining. Moreover, the number of TUNEL-positive cells was significantly higher in both the CD-STZ and WD-STZ groups than in the CD-con group. These results suggest that the effect of diabetes on the loss of SGNs may be stronger than that of a high-fat diet. Therefore, both STZ-treated groups may exhibit the greatest apoptosis progression. Interestingly, we also found elevated apoptosis cells in the SV of WD-con mice, which supported the metabolic hypothesis. Thus, both the degeneration of SGNs and cells in the SV contribute to the hearing impairment in those mice. The three parts of the cochlea that have been reported to be related to ARHL pathogenesis are the SGNs, SV, and hair cells. Considering the vascular supply system, the spiral ganglion is the most vulnerable area owing to the absence of collateral supply. The SV receives supply from adjacent branching arterioles when its single artery is blocked, while the organ of Corti is dually supplied from a direct supplying arteriole and labyrinthine fluid connecting all of the SV. Based on this structural difference and compensatory collateral systems, chronic ischemia sequentially affects the spiral ganglion and SV. This pattern was confirmed in this study.

In conclusion, we demonstrated that diabetes worsened ARHL in ApoE KO male mice fed with a WD through apoptosis of the SGNs and cells in the SV. Thus, hyperlipidemia and hyperglycemia can be good indicators for diagnosing the onset of hearing impairment. Furthermore, correction of them may avoid future occurrence of ARHL. We aim to analyze the correlation between diabetes and ARHL risk scores in the aged population to evaluate the findings in the human population in future studies.

## Data availability statement

The original contributions presented in the study are included in the article, further inquiries can be directed to the corresponding authors.

## Ethics statement

The animal study was reviewed and approved by Institutional Animal Care and Use Committee of Hallym University (Hallym2018-55).

## Author contributions

JS and JL were involved in the study concept and design. PN, YK, HS, and BK were involved in the acquisition of data. PN, HS, and BK were involved in the analysis and interpretation of data. PN, JL, and JS drafted the manuscript. CK was involved in the critical revision of the manuscript for important intellectual content. PN, CK, and HS were involved in the statistical analysis. BK and JS were involved in the technical and material support. JS and JL obtained funding. All authors had full access to all the data in the study and take responsibility for the integrity of the data and the accuracy of the data analysis. All authors contributed to the article and approved the submitted version.

## Funding

This work was supported by the National Research Foundation of Korea (NRF) grant funded by the Korea government (NRF 2016R1D1A1B01014128 and NRF-2016R1D1A2B02011858) and Korea Mouse Phenotyping Project (2014M3A9D5A01075129) of the Ministry of Science and ICT through the National Research Foundation, Republic of Korea.

## Conflict of interest

The authors declare that the research was conducted in the absence of any commercial or financial relationships that could be construed as a potential conflict of interest.

## Publisher's note

All claims expressed in this article are solely those of the authors and do not necessarily represent those of their affiliated organizations, or those of the publisher, the editors and the reviewers. Any product that may be evaluated in this article, or claim that may be made by its manufacturer, is not guaranteed or endorsed by the publisher.
